# Machine learning-enabled membership determination of open cluster AH03_J0748-26.9 using Gaia DR3 astrometry

**DOI:** 10.1038/s41598-026-67279-2

**Published:** 2026-08-25

**Authors:** W. A. Badawy, H. I. Abdel Rahman

**Affiliations:** Astronomy Department, National Research Institute of Astronomy and Geophysics, Helwan, Cairo, 11421 Egypt

**Keywords:** Galaxy, Open clusters and associations, Individual, AH03_J0748-26.9 — stars, Statistics — techniques, Photometric — techniques, Astrometric — catalogs, Gaia DR3, Astronomy and planetary science, Mathematics and computing, Optics and photonics, Physics

## Abstract

As populations of stars born concurrently, open clusters serve as fundamental laboratories for validating models of star formation, stellar evolution, and the dynamical processes of the Galactic disk. This paper provides a comprehensive astrometric and photometric analysis of the open cluster AH03_J0748-26.9, leveraging data from the Gaia DR3 catalog. We employ the Hierarchical Density-Based Spatial Clustering of Applications with Noise (HDBSCAN) algorithm to analyze astrometric data from Gaia Data Release 3 (DR3). Our analysis characterizes the identified clusters through several fundamental parameters, specifically the census of probable members, spatial radii, age, distance, the initial mass function (IMF), and total cluster mass.We determined a concentration parameter of *c=0.396 ± 0.077* for AH03_J0748-26.9. The estimated number of members for AH03_J0748-26.9 is 128 stars. A distance modulus of *13.427 ± 0.093* mag and an age of 114.815 Myr ($\log t=8.06 \pm 0.020$) were derived using PARSEC isochrone fitting. Given a relaxation time of 32.125 Myr and an age of 114.815 Myr (*τ = 3.57 > 1*), the cluster AH03_J0748-26.9 is found to be dynamically relaxed. We analyzed the mass function (MF) for AH03_J0748-26.9 using a Monte Carlo method coupled with synthetic cluster modeling. We characterized the MF over the range 0.102–$5.088\,M_\odot$ by fitting a two-segment broken power law, deriving slopes of $\alpha _A = -3.000 \pm 0.605$ (for $M > 0.15\,M_\odot$) and $\alpha _B = 1.596 \pm 0.183$ (for $M < 0.15\,M_\odot$) with a characteristic break mass of $M_C = 0.150 \pm 0.025\,M_\odot$. The steep slope in the higher mass regime suggests a deviation from the canonical Salpeter IMF, which is consistent with the effects of dynamical evolution and mass segregation. By integrating this best-fit MF and adopting a binary fraction of *∼ 0.70*—typical for concentrated open clusters—we estimate a total cluster mass of $290.603 \pm 3.605\,M_{\odot }$. A sensitivity analysis indicates that varying the binary fraction between 0.5 and 0.9 alters the total mass estimate by approximately $\pm 10\%$, supporting the robustness of the mass scale. A sample of 128 member stars yielded a mean radial velocity of $69.745\pm 9.615\ \text {km}\ \text {s}^{-1}$. These kinematic data, combined with proper motions, served as input for the galpy package to determine the cluster’s Galactic orbit.

## Introduction

Open clusters serve as fundamental tracers of the Milky Way’s disk evolution, as their ages span the full history of the Galaxy^1,2^. While older open clusters probe the thick disk, younger ones are primarily found within the perturbed thin disk and its spiral arms. Analyzing their kinematics and spatial distribution allows us to constrain the Galactic gravitational potential and the tidal forces shaping it. The dynamical evolution of open clusters is governed by internal stellar interactions and external tidal perturbations from the Galactic disk and molecular clouds^3,4^. Leveraging astrometric and photometric data, open clusters yield fundamental parameters – such as distance, age, metallicity, and mass – that are inaccessible from isolated stars, making them essential for reconstructing the formation and evolutionary history of the Galactic disk.

A persistent observational challenge, however, is the reliable separation of genuine cluster members from the dense foreground and background field population, which is critical for accurate parameter derivation. The advent of the Gaia mission, coupled with the refinement of sophisticated clustering techniques, has fundamentally transformed our understanding of stellar populations and the dynamics of open clusters. Widely adopted methods for identifying cluster members include UPMASK^5,6^, SHiP^7^, pyUPMASK^8^, and OCfinder^9^. These methodologies leverage a diverse array of clustering techniques–ranging from K-means and friends-of-friends to density-based approaches like DBSCAN and HDBSCAN–to isolate stellar systems with high efficiency. The most extensive catalog combining automated identification with Gaia DR3 data is the Unified Cluster Catalog^10^, comprising over 16,000 objects from 32 databases spanning 11 years. An effective algorithm for membership determination must satisfy three fundamental criteria: relative independence from the cluster’s morphology, accounting for variations in local stellar density, and computational efficiency for large-scale datasets^11^.

The open cluster AH03_J0748–26.9 (also known as FSR 1315 and MWSC 1347/1348) serves as a compelling case study for dedicated analysis. A review of existing literature reveals significant discrepancies regarding its fundamental parameters, motivating a re-evaluation using advanced machine learning techniques. For instance, previous surveys report varying member counts:^12^ identified 68 likely members, while^13^ utilized only 65 stars. Consequently, distance estimates range widely from approximately $3.47\,\textrm{kpc}$ to $4.11\,\textrm{kpc}$, and age estimates show variance ($\log t \approx 7.8$–8.06). These inconsistencies suggest that the cluster’s true stellar population may have been undersampled in previous automated sweeps, potentially biasing the derived physical parameters. Furthermore, AH03_J0748–26.9 is positioned in the outer Galactic disk ($R_{\textrm{GC}} \approx 11\,\textrm{kpc}$). Clusters in these outer regions experience different tidal shear forces compared to inner disk clusters, yet their dynamical evolution remains less characterized.

The specific scientific questions driving this work are: (1) Can a dedicated machine learning approach recover a more extensive and robust membership list to resolve the existing parameter discrepancies? (2) What are the dynamical state and mass distribution of this cluster, and do they show evidence of mass segregation or dynamical relaxation? This paper provides a comprehensive astrometric and photometric analysis of AH03_J0748–26.9 to address these questions. We employ the HDBSCAN algorithm on *Gaia* DR3 data to maximize member recovery. We then derive fundamental parameters, including the mass function and total mass, to investigate the cluster’s evolutionary status. Our analysis suggests that AH03_J0748–26.9 is likely a dynamically relaxed system with a distinct mass function, offering potential new insights into the evolution of open clusters in the outer Galactic disk.

The subsequent sections of this paper are structured as follows. Section “Data and membership determination” details the data and the membership determination process, introducing the HDBSCAN algorithm (“HDBSCAN algorithm”) and the resulting membership analysis (“Membership selection and HDBSCAN clustering”). Section “Physical properties and structural parameters” presents the basic derived parameters of the clusters, including their centers, radii (“Determination of cluster center and radius”), as well as galactic coordinates, ages, and distances (“Galactic distribution, evolutionary age, and distance estimates”). Section “Estimation of mass function of AH03_J0748-26.9” provides an analysis of the cluster’s mass function alongside an investigation into the orbital dynamics of AH03_J0748-26.9. Finally, Sect. “Conclusion” provides the summary and conclusions of this work.

## Data and membership determination

**Fig. 1:**
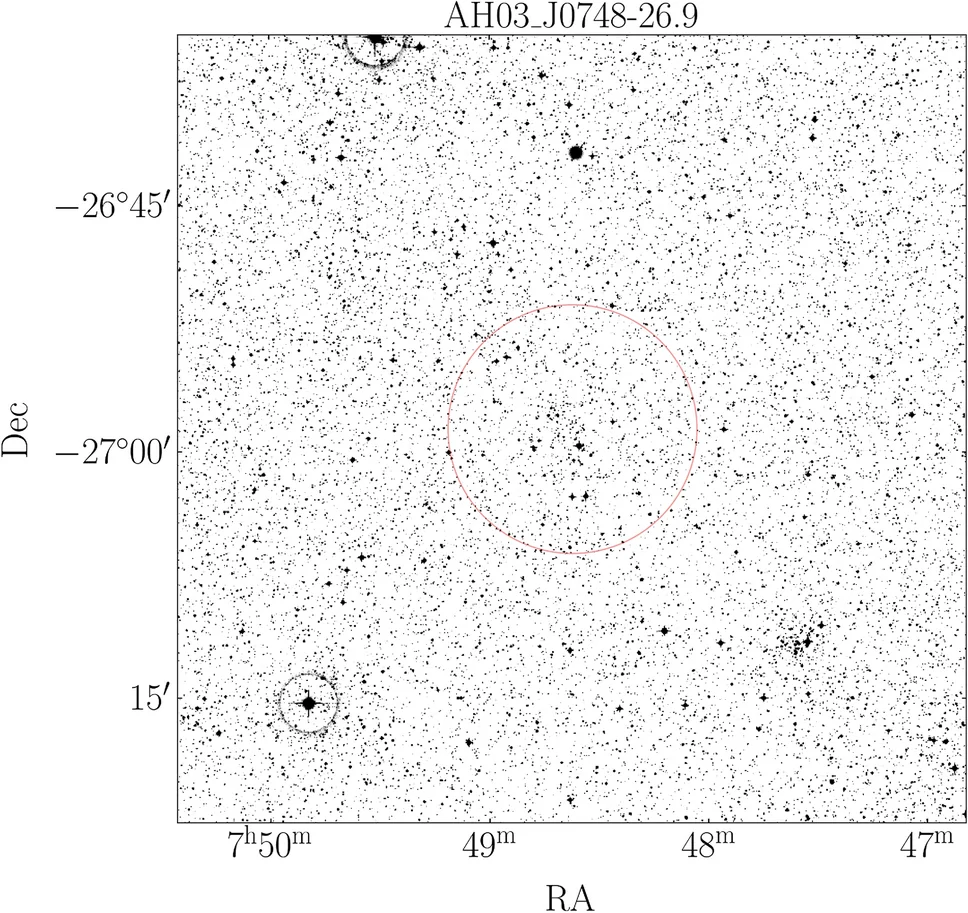
The cluster’s finding chart was sourced from the Digitized Sky Survey (DSS), with the center indicated by a red circle based on^12^.

Building upon its 2013 launch, the European Space Agency’s Gaia mission has provided its most comprehensive catalog to date through Data Release 3^14^, offering unprecedented astrophysical characterization for over 1.8 billion sources. The Gaia DR3 catalog comprises *G*-band photometry for roughly 1.81 billion sources, with a subset of 1.54 billion objects containing broad-band color information via $G_{\textrm{BP}}$ and $G_{\textrm{RP}}$ measurements^14^. The Gaia astrometric solution for each source is defined by five primary parameters: right ascension R.A. (*α *), decl. declination (*δ *), parallax (*ϖ *), and the proper motion components ($\mu _{\alpha }^{*},\mu _{\delta }$). Following standard convention, the longitudinal proper motion includes the cosmic correction factor, $\mu _{\alpha }^{*}=\mu _{\alpha }\cos \delta$. Furthermore, Gaia DR3 provides median radial velocities for over 33 million sources with $G_{\textrm{RVS}}<14$ mag, while integrated photometry ($G,G_{\textrm{BP}},G_{\textrm{RP}}$) is available for approximately 1.5 to 1.8 billion cataloged objects^14^. The Gaia DR3 catalog encompasses sources spanning a wide dynamic range, from the bright limit at *G≈ 3* mag to the faint observational threshold near *G≈ 21* mag. The finding chart, sourced from the Digitized Sky Survey (DSS), is presented in Fig. 1, with the cluster centre indicated by a red circle. For the initial phase of our analysis, we adopt the structural parameters for AH03_J0748-26.9 reported by^12^, specifically utilizing their determination of the cluster’s central coordinates. Astrometric and photometric data for AH03_J0748-26.9 were retrieved from the Gaia DR3 archive via the astroquery.gaia Python module https://astroquery.readthedocs.io/en/latest/gaia/gaia.html. We selected all sources within a $0.5^{\circ }$ angular radius of the cluster center, applying specific quality filters to ensure data integrity. Using the astroquery package^15^, we queried the Gaia Archive https://gea.esac.esa.int/archive/ to retrieve a comprehensive astrometric and photometric dataset. For each source, we obtained celestial coordinates $(\alpha ,\delta )$, proper motions $(\mu _{\alpha }^{*},\mu _{\delta })$, and parallaxes (*ϖ *), alongside $G,G_{\textrm{BP}},$ and $G_{\textrm{RP}}$ magnitudes, radial velocities (RV), and their associated uncertainties. Our analysis incorporates both astrometric and photometric components, specifically leveraging *G*-band magnitudes and $G_{\textrm{BP}}-G_{\textrm{RP}}$ colors to characterize the cluster population.

To ensure the fidelity of the astrometric data, we applied a rigorous set of quality filters to the initial dataset. We primarily filtered the sample based on the Renormalized Unit Weight Error (RUWE), excluding sources with $\textrm{RUWE} > 1.4$, as values exceeding this threshold typically indicate poor fits to the single-star model^16^. In addition to RUWE, we applied a filter on the astrometric excess noise , requiring $\texttt {astrometric\_excess\_noise} < 1.0 \,\,\textrm{mas}$ to remove sources with significant excess noise indicative of problematic astrometric solutions (e.g., unresolved binaries or extended sources). Regarding the relative parallax error, we opted not to impose a strict signal-to-noise ratio cut (e.g., $\texttt {parallax\_over\_error} > 10$). This decision was made to avoid truncating the cluster sequence at the faint end, relying instead on the HDBSCAN algorithm’s ability to distinguish cluster populations from field stars within the 5D phase space. These combined quality criteria effectively eliminated sources with poor astrometric solutions while retaining valid detections down to the survey’s faint limit of $G \sim 21\,\textrm{mag}$.

### HDBSCAN algorithm

To isolate the stellar population of AH03_J0748-26.9 from the Galactic field, we employed the Hierarchical Density-Based Spatial Clustering of Applications with Noise (HDBSCAN) algorithm^17^. This unsupervised learning approach allows for the identification of cluster members based on their local density in multidimensional phase space. The selection of HDBSCAN is motivated by its high recovery rate;^18^ demonstrated that the algorithm achieves a sensitivity of up to 82%, maintaining robust performance across the diverse distances and scales characteristic of the Galactic open cluster population.

HDBSCAN is well-suited for Galactic studies as it accommodates clusters of non-uniform morphology and varying densities. Unlike centroid-based methods, it does not require a prior specification of the number of clusters, though the final membership selection remains dependent on the chosen hyperparameters, particularly the minimum cluster size. HDBSCAN^17^ extends the DBSCAN framework by incorporating hierarchical clustering principles, allowing it to identify stellar associations across a range of densities–a capability essential for mapping the full extent of AH03_J0748-26.9 without artificially truncating the lower-density outer regions.

Implemented in Python by^19^, this version of HDBSCAN has become a standard tool in both data science and astrophysical research due to its computational efficiency and robust performance. The HDBSCAN implementation utilizes a density-based framework to construct a hierarchical representation of the dataset, characterizing clusters with irregular geometries while maintaining separation between genuine members and background noise. By integrating hierarchical clustering principles, HDBSCAN enhances the detection of complex density variations within the Gaia dataset, providing a more nuanced identification of stellar groupings compared to traditional flat-clustering methods, which often struggle with non-uniform field densities.

One of the primary advantages of HDBSCAN is its streamlined parameterization: it essentially relies on a single hyperparameter, the minimum cluster size ($m_{\textrm{clSize}}$), which dictates the smallest grouping that the algorithm will consider a legitimate cluster. Adopting an overly restrictive $m_{\textrm{clSize}}$ tends to produce a highly fragmented cluster hierarchy, increasing sensitivity to small-scale structures but risking over-segmentation into numerous statistically insignificant groupings. Conversely, an excessively large $m_{\textrm{clSize}}$ may lead to the artificial merging of distinct sub-structures within the hierarchical tree. In our analysis, we adopted $m_{\textrm{clSize}}=80$ to ensure the identification of a statistically significant stellar population. To enhance the recovery of sparse features within the cluster, we utilized the ’leaf’ selection method^19^, which identifies clusters at the bottom of the hierarchical tree rather than merging them based on global stability. Furthermore, following the recommendations of^17^, we set the smoothing parameter $m_{\textrm{pts}}=10$ to optimize both the algorithm’s sensitivity and computational performance.

### Membership selection and HDBSCAN clustering

The spatial domain for the HDBSCAN membership analysis was centered on the nominal coordinates of the AH03_J0748–26.9 open cluster. Utilizing a 5D feature space comprising $(\alpha , \delta , \mu _{\alpha ^{*}}, \mu _{\delta }, \varpi )$, we applied the HDBSCAN algorithm to stars within a $0.6^{\circ }$ radius of the AH03_J0748–26.9 cluster center ($\alpha = 117.155^{\circ }, \delta = -26.972^{\circ }$). Regarding feature scaling, the input parameters were used in their original *Gaia* DR3 units: degrees for positions (RA, Dec), mas yr$^{-1}$ for proper motions, and mas for parallax. Prior to clustering, all input features were standardized using the StandardScaler routine from the scikit-learn Python library, transforming each parameter to zero mean and unit variance. This step is essential when combining astrometric quantities expressed in different physical units – degrees for positions (R.A., Decl.), mas yr$^{-1}$ for proper motions, and mas for parallax – as it prevents parameters with larger numerical ranges from dominating the density estimation and ensures that HDBSCAN operates on a balanced, physically meaningful feature space. To ensure the reliability of the identified members for AH03_J0748–26.9, we performed a sensitivity analysis on the HDBSCAN hyperparameters. We executed the algorithm across five different settings for the minimum cluster size (min_cluster_size): 10, 20, 40, 60, and 80, while keeping the minimum samples parameter min_samples fixed at 10. We observed that the cluster population remained highly stable for $\texttt {min\_cluster\_size} \ge 20$. The final membership determination was based on the run with min_cluster_size *= 80*, which provides a conservative estimate of the cluster population by filtering out smaller, potentially spurious density fluctuations. Membership probabilities (*P*) were derived directly from the probabilities_ attribute, which quantifies the likelihood of a star belonging to the identified cluster based on the distance to the cluster’s density core relative to the noise. An analysis of AH03_J0748–26.9’s membership identifies 128 probable member stars (*P > 0.5*), with 112 being highly probable (*P > 0.9*) and 82 being very highly probable (*P > 0.99*).

**Fig. 2:**
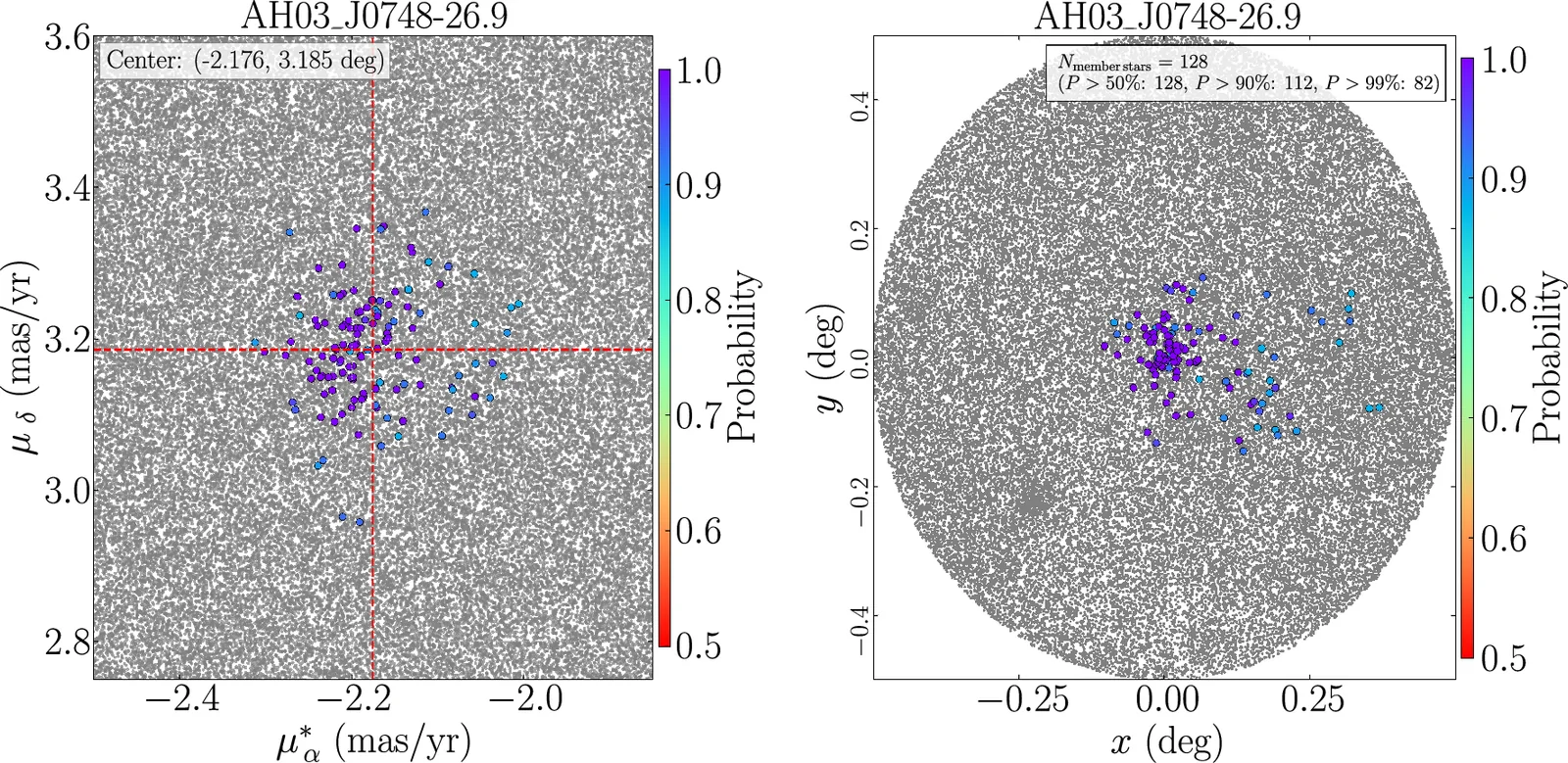
The color bar on the right-hand side represents the membership probabilities of stars within the VPDs for AH03_J0748-26.9, derived from the Gaia DR3 astrometric catalog.

To illustrate the kinematic properties of the most probable members within $AH03\_J0748-26.9$, we generated vector-point diagrams (VPDs) as displayed in Fig. 2.The mean proper-motion components for the cluster were derived from the ensemble of high-probability members, as illustrated in Fig. 2. The mean proper-motion components for AH03_J0748-26.9 were determined to be $(\mu _{\alpha }^{*},\mu _{\delta })=(-2.176\pm 0.005,3.185\pm 0.006)$ mas $\textrm{yr}^{-1}$ . The derived mean proper-motion components for AH03_J0748-26.9 are summarized in Table 2.

**Fig. 3:**
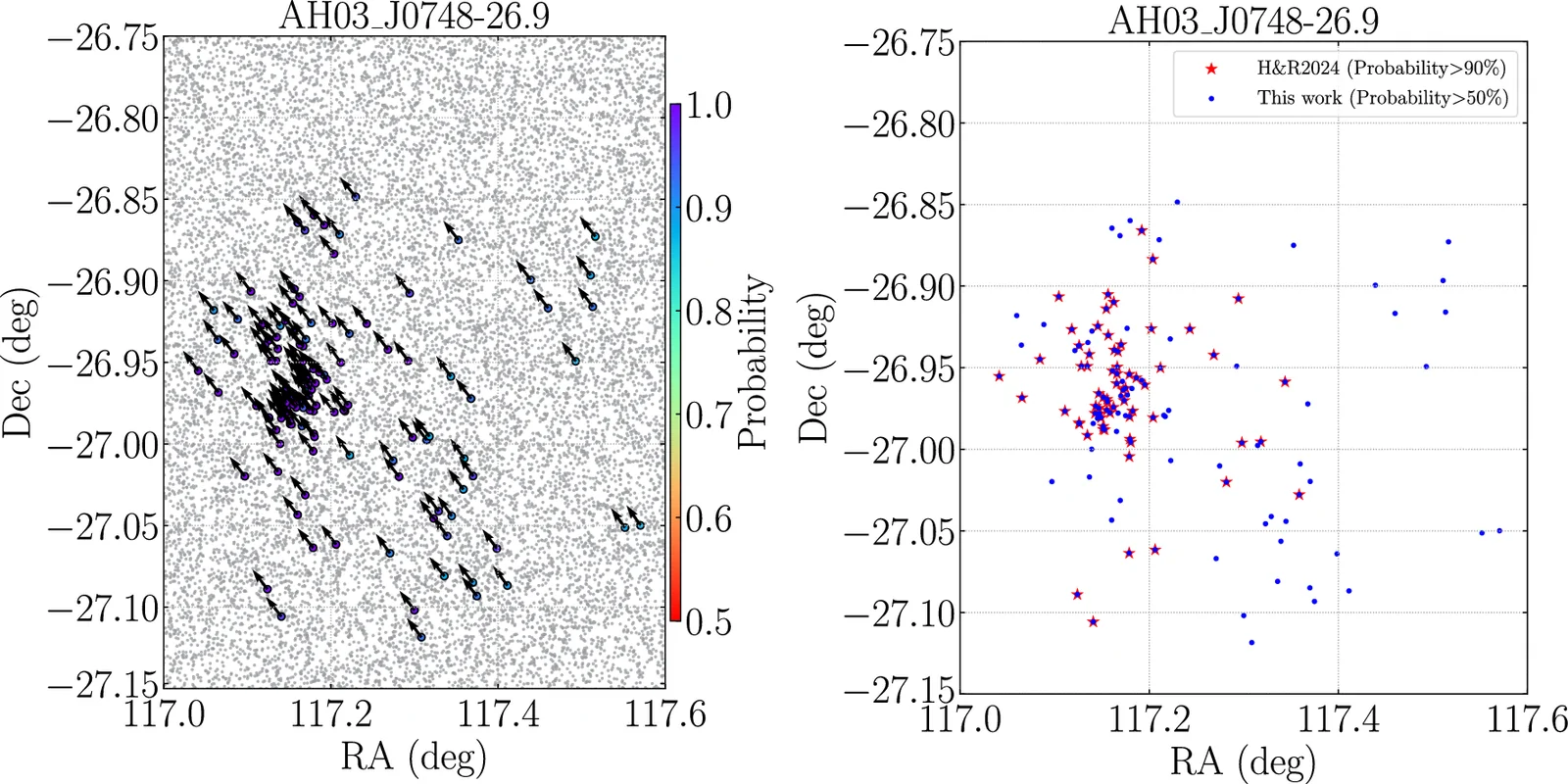
Left panel: Co-moving stellar distribution of AH03_J0748-26.9. Black arrows indicate member stars (membership probability *P > 0.5*); gray points represent field stars. Right panel: Spatial distribution of member stars identified in this work (blue points, *P > 0.5*) compared with members reported by Hunt & Reffert (2024^12^, red stars). The *P > 0.5* threshold is applied uniformly to our sample because it represents the nominal membership criterion adopted throughout this study. This direct comparison illustrates that our HDBSCAN-based method recovers a larger and more spatially extended stellar population than previous catalogs.

Figure 3 (left panel) displays the proper motion vectors $(\mu _{\alpha }\cos \delta ,\mu _{\delta })$ relative to the celestial coordinates $(\alpha ,\delta )$ for the identified cluster stars. The high degree of alignment in these vectors suggests that the member stars share a common space velocity. This uniformity supports the reliability of the cluster membership determination and highlights the kinematic coherence of the group.

**Fig. 4:**
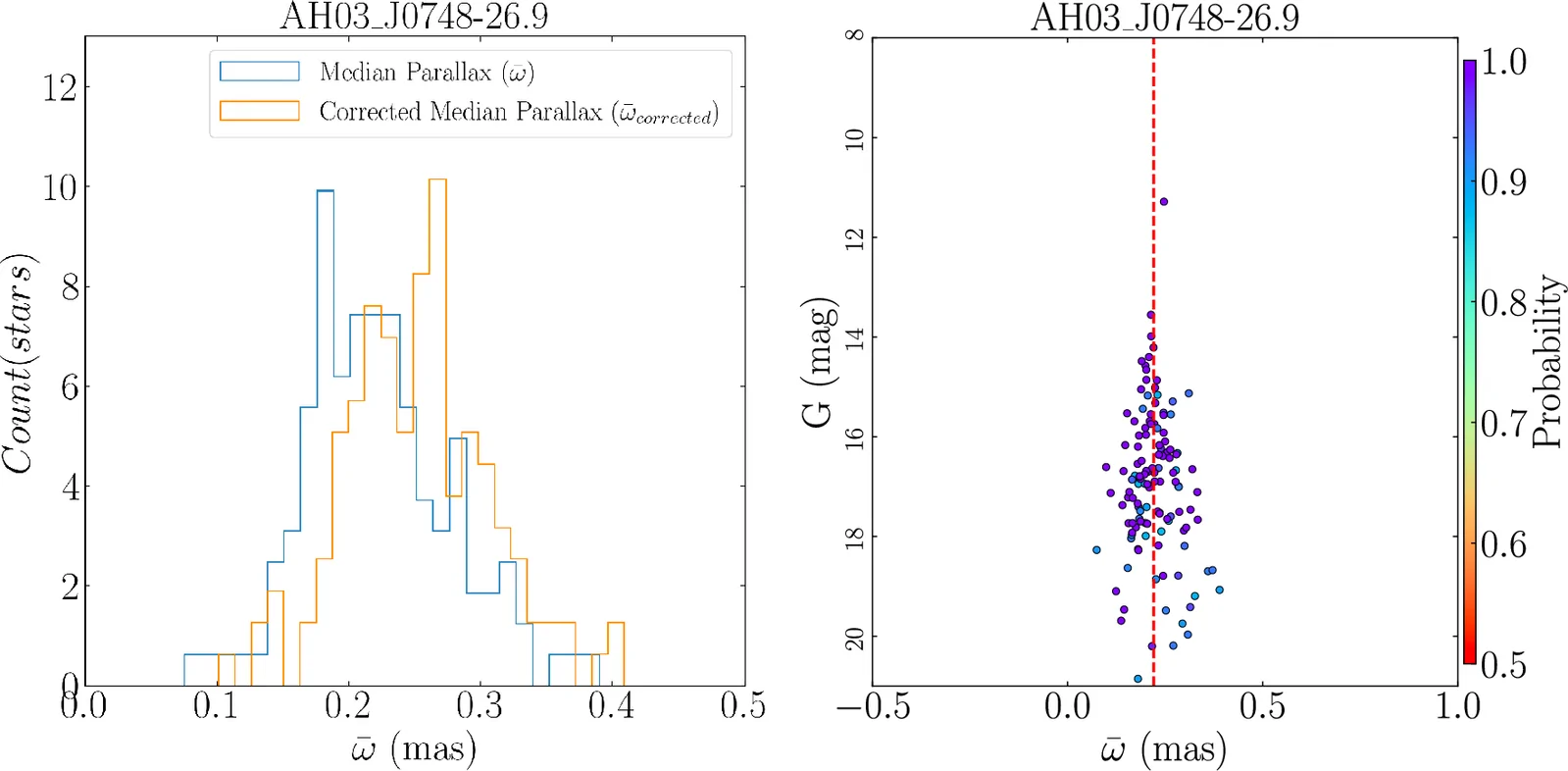
Parallax distribution for AH03_J0748-26.9 derived from Gaia DR3 without applying a relative parallax error constraint. Left panel: Histogram of parallaxes for the 128 probable member stars (membership probability *P > 0.5*). Right panel: *G*-magnitude versus parallax diagram showing only the probable cluster members, color-coded by their membership probability (darker colors indicate higher probabilities). The median parallax after zero-point correction ($\bar{\omega } = 0.236 \pm 0.005$ mas) is marked by the vertical dashed line in the left panel and the horizontal solid line in the right panel.

To validate our membership selection against prior work, Fig. 3 (right panel) compares the spatial distribution of our adopted members (probability *P > 0.5*) with those reported by Hunt & Reffert (2024)^12^ from their catalog. The *P > 0.5* threshold is used consistently because it constitutes our nominal membership criterion throughout this study. This fixed threshold provides a direct, unbiased visual assessment of whether the HDBSCAN algorithm recovers a more extended or numerous cluster population compared to previous methods, without introducing additional degrees of freedom from threshold variation. The comparison demonstrates that our approach identifies a larger and more spatially extended member sample, particularly in the cluster outskirts. The median trigonometric parallax (*ϖ *) for AH03_J0748-26.9 was derived from a histogram analysis of the highest-probability cluster members (see Fig. 4). In accordance with the Gaia DR3 documentation, we applied the parallax zero-point corrections using the official source code https://gitlab.com/icc-ub/public/gaiadr3_zeropoint provided by^20^. Following the zero-point correction, the median parallax for AH03_J0748-26.9 was calculated as *0.236± 0.005* mas. Using the standard relation $d\text { (pc)}=1000/\varpi \text { (mas)}$, we derived a heliocentric distance of $d_{\varpi }=4.834\pm 0.127$ kpc for AH03_J0748-26.9. Fig. 4 illustrates the relationship between *G* - magnitude and parallax, with probable cluster members color-coded by their membership probability.

**Fig. 5:**
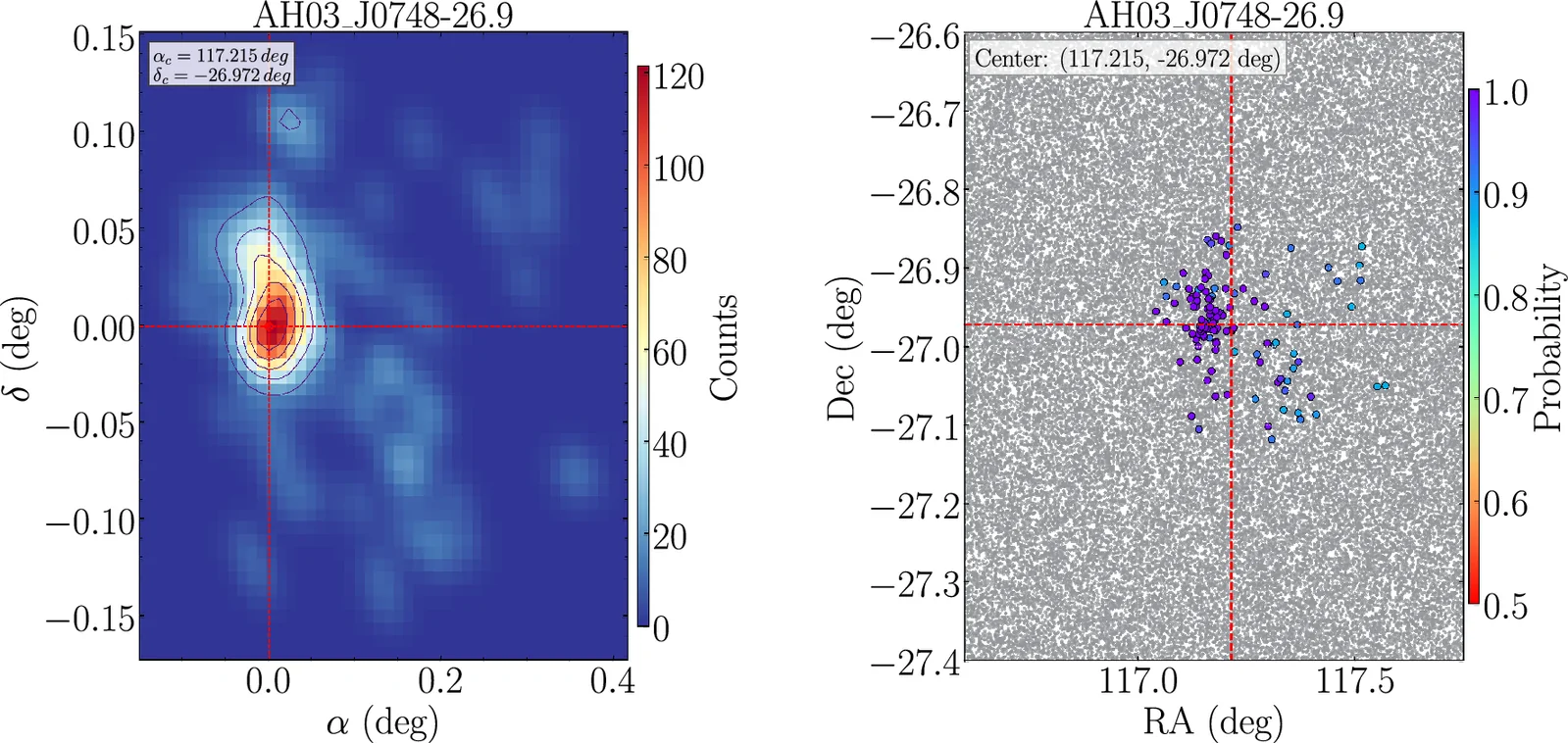
The spatial distribution of the studied cluster is presented in the contour map, with the intersection of the dashed red lines pinpointing the mean stellar coordinates. Spatial distribution of stellar candidates in the direction of AH03_J0748-26.9. High-probability members (*P \ge 0.5*) are indicated by the color-mapped points, where the color bar represents the membership probability derived from the selection algorithm. The dashed red lines indicate the calculated mean center of the cluster under study at $(\alpha , \delta ) = (117.215^\circ , -26.972^\circ )$.

## Physical properties and structural parameters

### Determination of cluster center and radius

Open clusters are characterized by their loosely bound nature and sparse stellar distribution, which nevertheless converges into a well-defined density peak at the center. To achieve a meticulous determination of the cluster center, we calculated the mean right ascension (*α *) and declination (*δ *) of the identified members, as illustrated in Fig. 5. The central coordinates for AH03_J0748-26.9 were estimated to be $(\alpha ,\delta )=(117.215\pm 0.01{}^{\circ },-26.972\pm 0.005{}^{\circ })$. The spatial distribution of the cluster is illustrated in the contour map (Fig. 5), where dashed green lines mark the intersection of the mean stellar positions. We examined a spatial area significantly larger than the apparent visual diameter of the cluster to ensure an accurate determination of its structural limits and to investigate the presence of an extended stellar corona. To achieve this, we constructed the radial density profile (RDP) of AH03_J0748-26.9, allowing for a quantitative assessment of its structural parameters. We divided the cluster area into a series of concentric annuli with a constant width of 1 arcmin, subsequently calculating the stellar surface density within each ring. The radial density profile (RDP) remains a cornerstone methodology for characterizing the structural properties and spatial morphology of open clusters. The stellar surface density within each concentric annulus is calculated by dividing the number of stars by the area of the ring, as expressed in Eq. (1):

1 $$\begin{aligned} \rho _{i}=\frac{N_{i}}{\pi \left( r_{i+1}^{2}-r_{i}^{2}\right) }. \end{aligned}$$

**Fig. 6:**
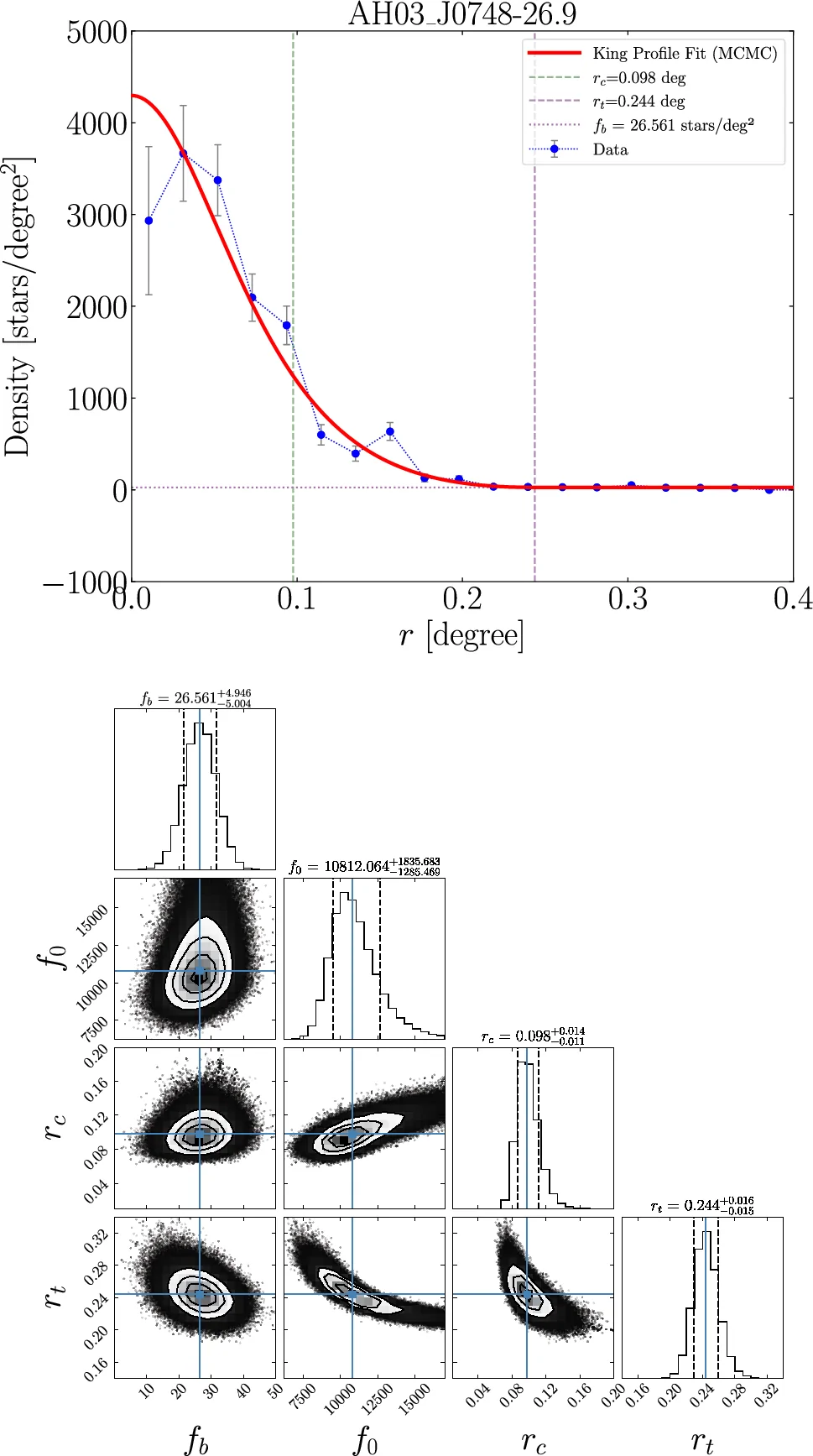
The structural parameters for cluster AH03_J0748-26.9 are presented, featuring a King model fit to the radial density profile in the upper panel and the corresponding MCMC-derived marginalized posterior distributions in the lower panel.

In this expression, $N_{i}$ denotes the count of member stars located within the *i*-th annulus, which is defined by an inner boundary $r_{i}$ and an outer boundary $r_{i+1}$. The resulting radial density profile is presented in Fig. 6, illustrating the decline in stellar density as a function of distance from the cluster center.

To quantitatively characterize the cluster structure, we fitted the observed RDP with a King (1962)^21^ analytical profile, represented by the blue curve in Fig. 6. The model is defined as Eq. (2):

2 $$\begin{aligned} f(r)=f_{\text {b}}+f_{0}\left( \frac{1}{\sqrt{1+\left( \frac{r}{{r_{c}}}\right) ^{2}}}-\frac{1}{\sqrt{1+\left( \frac{{r_{t}}}{{r_{c}}}\right) ^{2}}}\right) ^{2}. \end{aligned}$$

In this expression, $f_{\textrm{b}}$ represents the background density, and $f_{0}$ acts as a normalization coefficient that scales the amplitude of the cluster’s density profile. Due to the finite tidal radius, the actual central surface density is given by

$$ f(0) = f_{\textrm{b}} + f_{0} \left[ 1 - \left( 1 + \left( \frac{{r_{\textrm{t}}}}{{r_{\textrm{c}}}}\right) ^{2} \right) ^{-1/2} \right] ^{2}. $$

The parameter $r_{\textrm{c}}$ corresponds to the core radius, classically defined as the distance from the cluster center at which the surface density drops to half of its central value (above the background), whereas $r_{\textrm{t}}$ represents the tidal radius, defined as the radial distance at which the cluster density merges with the field density (i.e., $f(r_{\textrm{t}}) = f_{\textrm{b}}$). The radial density profile was derived by calculating the angular separation of each *i*-th member $(\alpha _{i},\delta _{i})$ from the determined cluster center $(\alpha _{\text {cen}},\delta _{\text {cen}})$. The distance of each member *i* from the cluster’s geometric center was derived using the relation $r=\sqrt{(\Delta \alpha )^{2}+(\Delta \delta )^{2}}$, where *N* denotes the total number of stars in the sample. The uncertainty associated with the stellar density in each annulus was estimated based on Poisson statistics, where $\sigma _{i}=\sqrt{N_{i}}/A_{i}$. Here, $N_{i}$ represents the star count and $A_{i}$ the area of the *i*-th ring. The cluster’s structural extent is defined by its tidal radius ($r_{\textrm{t}} = 0.244 \pm 0.015~\textrm{deg} = 20.57~\textrm{pc}$, compared to $13.66~\textrm{pc}$ in^22^) and its core radius ($r_{\textrm{c}} = 0.098 \pm 0.013~\textrm{deg} = 8.26~\textrm{pc}$, compared to $1.12~\textrm{pc}$ in^22^). The core radius represents the distance at which the surface density drops to half of its peak central value ($f_{0}$). For AH03_J0748-26.9, we calculated a half-number radius ($r_{50}$) of $0.084^{\circ }$. The structural parameters derived from the King profile fitting for all studied clusters are summarized in Table 2.

To determine the structural parameters and their associated uncertainties, we employed a Bayesian Markov Chain Monte Carlo (MCMC) approach^23^, implemented via the emcee Python package^24^ . For the Bayesian inference, we configured the MCMC sampler with 2000 walkers, executing 1000 iterations per walker. To ensure the chains reached a steady state, the initial 200 steps were discarded as burn-in, following the procedures described by^25,26^. To ensure the trustworthiness of our Bayesian inference, we rigorously assessed the convergence and efficiency of the MCMC chains. We calculated the integrated autocorrelation time (*τ *) for each parameter to determine the spacing between independent samples. The initial burn-in phase was dynamically set to $5 \times \tau _{\max }$ to guarantee the exclusion of non-stationary transient steps. We computed the Effective Sample Size (ESS) as $N_{\textrm{walkers}} \times N_{\textrm{steps}} / \tau$, ensuring $\textrm{ESS} > 10{,}000$ for all parameters, which indicates robust estimates of the 16th, 50th, and 84th percentiles. Furthermore, we calculated the Gelman–Rubin $\hat{R}$ statistic (split-$\hat{R}$) across the walkers.

For all fitted parameters $(f_b, f_0, r_c, r_t)$, we found $\hat{R} = 1.000$, indicating that the chains have converged effectively to the target posterior distribution.

Our analysis reveals that the target cluster exhibits a significantly higher central stellar density compared to the surrounding Galactic field, indicating a highly concentrated core structure. Consequently, the high central concentration of the cluster suggests a robust gravitational binding, increasing the likelihood of its long-term survival against Galactic tidal forces. The lower panel of Fig. 6 displays the marginalized posterior distributions for the King profile parameters ($f_{0},{r_{c}},f_{b},{r_{t}}$), illustrating the likelihood of each value and the absence of strong degeneracies. The high degree of symmetry in the posterior distributions indicates that the mean and median values are nearly identical, following the behavior observed in^27^. Consequently, we adopted the 50th percentile as the best-fit parameter value.

To quantify the central condensation, we calculated the concentration parameter $c = \log _{10}(r_t / r_c)$ (King 1962; Peterson & King 1975)^21,28^. A high value indicates a dense core relative to the tidal boundary, typically associated with advanced dynamical evolution and mass segregation. For AH03_J0748-26.9, we obtained *c = 0.396 ± 0.077*.

### Galactic distribution, evolutionary age, and distance estimates

**Fig. 7:**
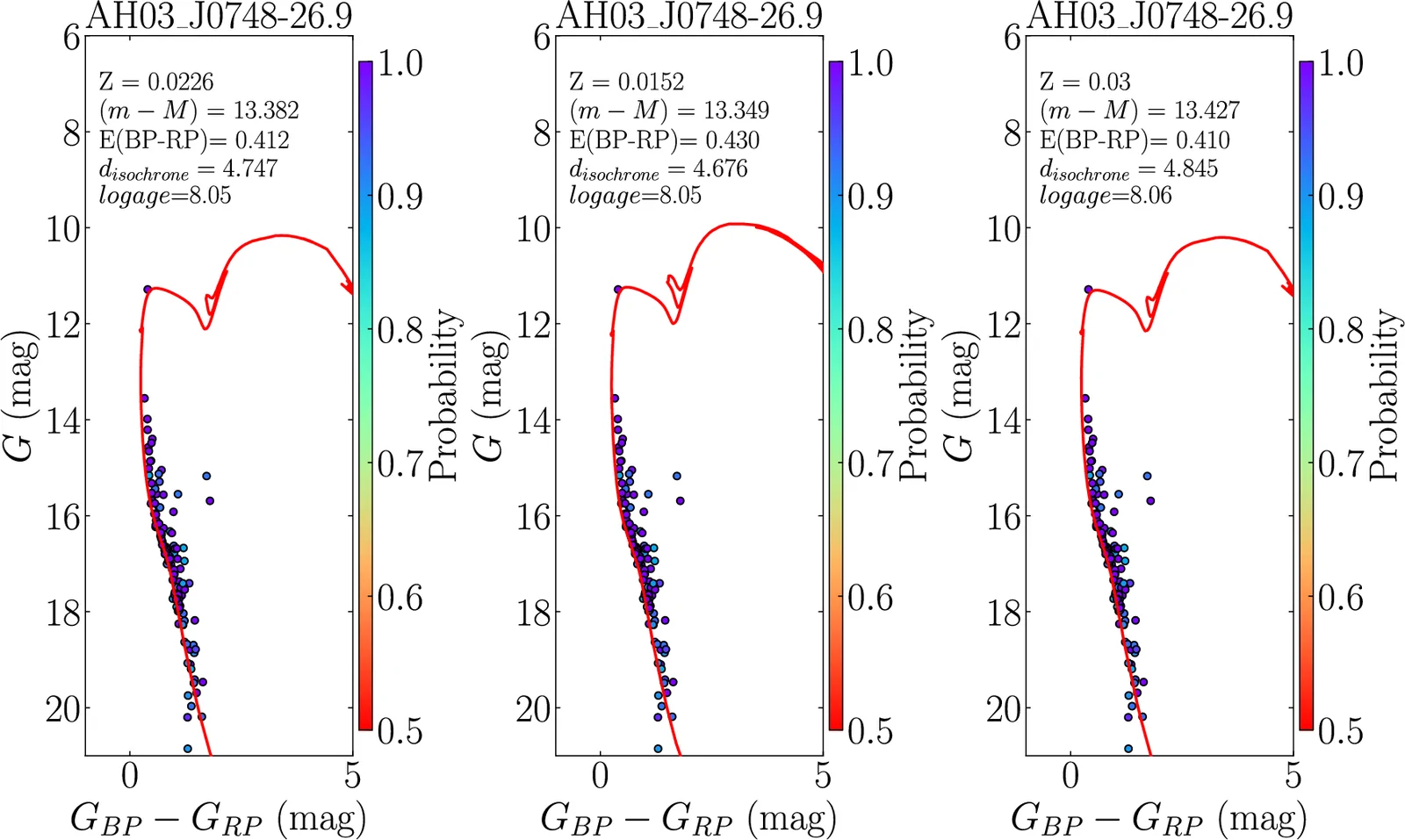
Color–magnitude diagrams (CMDs) for the AH03_J0748-26.9 cluster, derived from Gaia DR3 photometry. The data points are color-coded according to stellar membership probabilities, illustrating the distribution of likely cluster members along the evolutionary tracks.

We derived the clusters’ basic properties by matching observed color–magnitude diagrams with^29^ theoretical evolutionary isochrones. Based on the PARSEC database^30^, we computed isochrones at $Z = 0.0152,\ 0.0226,\ \text {and}\ 0.03$ for ages *łog (t) = 6.0*–10.0 in 0.05 dex steps. In this section, we used the magnitudes of stars in various filters to reliably determine the cluster properties through isochrone fitting. By performing a fit to the color–magnitude diagram (CMD) of the open cluster AH03_J0748-26.9, we identified the optimal matching isochrone. The observed color-magnitude diagrams (CMDs) of cluster members were analyzed using a $\chi ^{2}$ minimization routine to identify the best-fitting theoretical PARSEC isochrones. By minimizing the $\chi ^{2}$ statistic, this method compares observed Gaia magnitudes against theoretical models while incorporating photometric uncertainties. This process allows us to simultaneously derive the cluster’s age, distance modulus (*m-M*), and reddening *E(BP-RP)*. The fundamental parameters derived from the isochrone fitting process are summarized in Table 2, with the corresponding best-fit CMDs for the open clusters illustrated in Fig. 7. The uncertainties for the parameters derived in this section were estimated directly from the $\chi ^{2}$ minimization procedure. This section details the results of the isochrone fitting procedure and the physical parameters derived for the cluster under study. For the AH03_J0748-26.9 cluster, we constructed color–magnitude diagrams $(G,G_{\textrm{BP}}-G_{\textrm{RP}})$, which are illustrated in Fig. 7. Following an iterative fitting process across a range of ages and metallicities, we identified the best-fit model at *Z=0.03* and ($\log t=8.06 \pm 0.020$).

We note that the adopted metallicity *Z = 0.03* (slightly super-solar, $Z/Z_\odot \approx 1.97$ for $Z_\odot = 0.0152$) warrants discussion in light of the cluster’s Galactocentric location. At $R_{\textrm{GC}} \approx 11$ kpc, the Galactic radial metallicity gradient of approximately *-0.06* dex kpc$^{-1}$ (Donor et al. 2020^31^; Magrini et al. 2022^32^) predicts [Fe/H] *≈ -0.16* dex relative to solar, corresponding to *Z ≈ 0.011*. This is more consistent with our *Z = 0.0152* grid than with *Z = 0.03*. In the absence of spectroscopic observations, the metallicity of AH03–J0748−26.9 cannot be uniquely constrained from photometry alone, as the age, distance modulus, reddening, and metallicity are partially degenerate in the CMD. To assess the sensitivity of our results to this uncertainty, we fitted isochrones at three metallicities spanning *Z = 0.0152* to *Z = 0.03* (Fig. 7). The resulting distance moduli differ by only *Δ (m-M) = 0.078* mag, and all three fits converge on *łog (t) = 8.05*–8.06, well within the quoted uncertainties. Our principal conclusions regarding the cluster’s age, distance, and dynamical state are therefore robust across this metallicity range. We adopt *Z = 0.03* as the formally best-fitting solution to the observed CMD, while acknowledging that metallicities in the range *Z = 0.0152-0.0226* are physically motivated by the Galactic gradient and yield equally consistent results within the uncertainties of our fitting procedure. Thus, our conclusions regarding age and distance should be considered robust against metallicity uncertainties, but the true metallicity remains to be determined spectroscopically. Spectroscopic follow-up would be required to break the photometric degeneracy and determine the true metallicity of this cluster. The fitting procedure yields a distance modulus of *13.427 ± 0.093* mag. The best-fit isochrone analysis (at metallicity *Z=0.03* and ($\log t=8.06 \pm 0.020$) yields a photometric distance of $d_{\textrm{iso}} = 4.845 \pm 0.056$ kpc. This value is in close agreement with the parallax distance of $d_{\varpi }=4.834\pm 0.127$ kpc derived from Gaia DR3. To cross-validate, we applied the Bayesian geometric distance estimator of Bailer-Jones et al. (2021)^33^ to the cluster’s median parallax, obtaining $d_{\textrm{BJ}} = 4.839 \pm 0.102$ kpc. The three independent estimates are mutually consistent within *1σ *. Given the higher precision of the isochrone fit and its consistency with the cluster’s color-magnitude diagram morphology, we adopt $d_{\textrm{iso}} = 4.845 \pm 0.056$ kpc as the final distance for AH03_J0748-26.9 throughout this work. Furthermore, we determined the color excess to be $E(G_{\textrm{BP}}-G_{\textrm{RP}})=0.410 \pm 0.050$ mag, which corresponds to *E(B-V)=0.306 ± 0.037* mag.

**Fig. 8:**
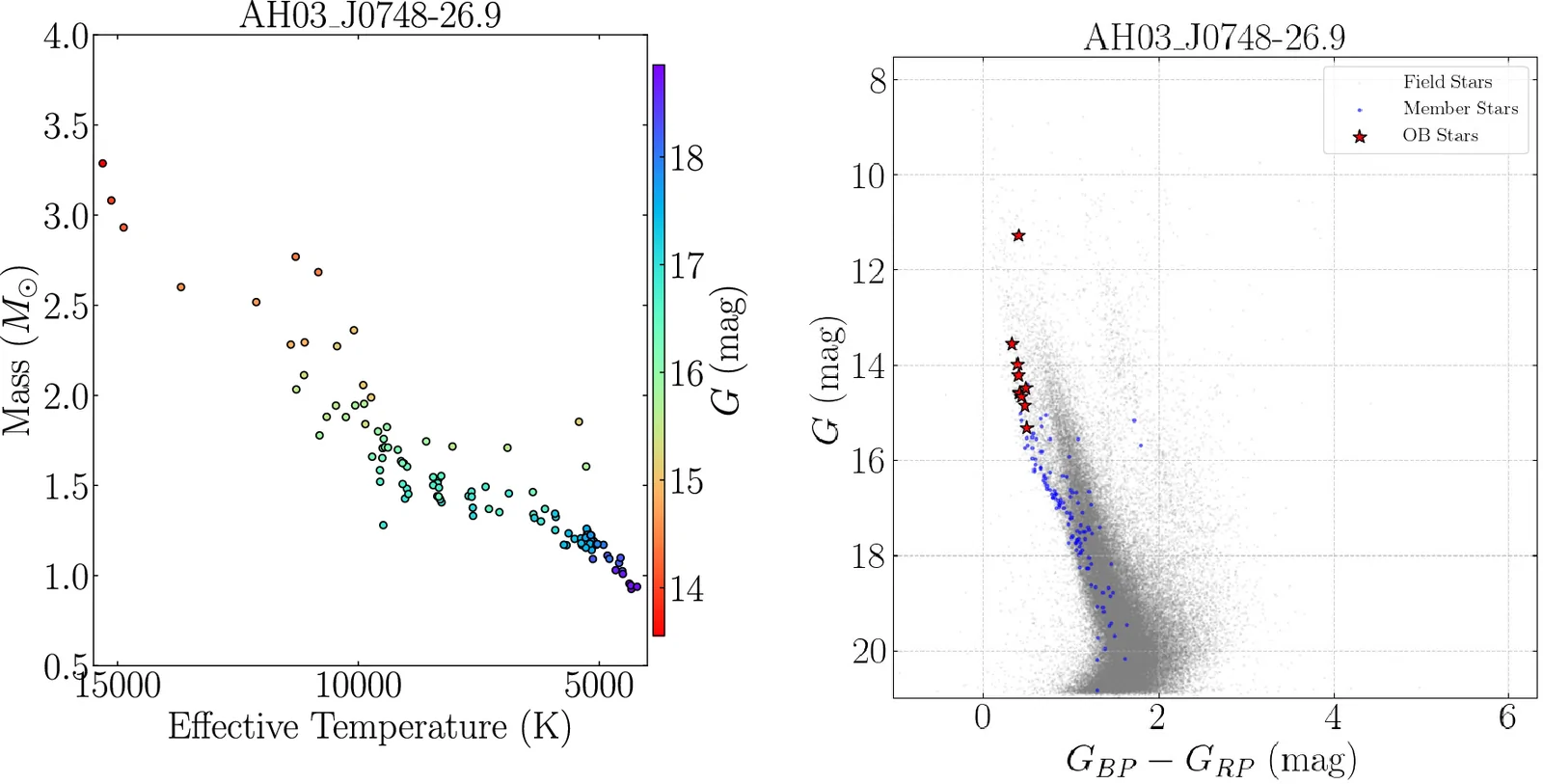
Color-Magnitude Diagram (CMD) for the region surrounding AH03_J0748-26.9. The gray points represent the ambient field population, providing a background context. Blue points delineate the identified cluster member stars, revealing a clear main sequence. The prominent red stars indicate the high-mass OB population, highlighting the cluster’s most luminous and massive components. Magnitudes are shown in the Gaia *G* band against the $G_{BP}-G_{RP}$ color index. Mass-Temperature relationship for members of AH03_J0748-26.9. The x-axis is inverted to follow standard astrophysical convention, placing higher effective temperatures ($T_{eff}$) on the left. Data points are color-coded by Gaia *G* magnitude, where lighter colors indicate brighter stars. The plot highlights the concentration of stellar mass relative to atmospheric heating within the cluster.

To provide context for the upper main sequence population, we cross-matched our member list with the catalog of ^34^. This catalog estimates effective temperatures ($T_{\textrm{eff}}$) from *Gaia* colors, which are then used to assign nominal spectral types. We identified nine probable B-type members (nominal spectral types B4–B9) based purely on these photometric $T_{\textrm{eff}}$ estimates, as detailed in Table 1. These objects possess derived masses ranging from 2–$5\,M_{\odot }$ and photometric temperatures between 11, 600 K and 16, 800 K. As shown in Fig. 8, these stars trace the blue envelope of the main sequence. While this identification relies entirely on photometry rather than spectroscopic classification, the presence of these massive stars qualitatively supports the derived isochrone age of $\log t = 8.06 \pm 0.020$, as such massive stars would have evolved off the main sequence in older systems. The mass–temperature distribution of the cluster members, shown in the left panel of Fig. 8, further illustrates the distribution of the upper main sequence. The color excess has been converted to *E(B-V)*, and the magnitudes have been corrected for interstellar reddening using the relation $A_{V}=R_{V}E(B-V)$, with $R_{V}=3.1$. Following the Gaia passband definitions provided by^35^ , we determined the extinction in the *G* band ($A_{G}$) using the color excess relations from^36^. Specifically, $A_{G}$ is calculated as:

$$ A_{G}=1.89\times E(G_{\textrm{BP}}-G_{\textrm{RP}}). $$

This allows for a direct correction of the observed magnitudes based on the derived reddening. The individual extinctions were computed as:

3 $$\begin{aligned} A_{G_{\text {BP}}} = 1.083 A_V, \quad A_{G_{\text {RP}}} = 0.634 A_V, \quad A_G = 0.836 A_V. \end{aligned}$$

From these, the following transformations were derived:

4 $$\begin{aligned} A_V = 2.227 E(G_{\text {BP}} - G_{\text {RP}}), \quad A_G = 1.862 E(G_{\text {BP}} - G_{\text {RP}}). \end{aligned}$$

Based on the clusters’ distances, longitudes (*l*), and latitudes (*b*), we calculated their Galactocentric coordinates. In this system, $X_{\odot }$ is directed toward the Galactic center, $Y_{\odot }$ follows the direction of Galactic rotation, and $Z_{\odot }$ indicates the vertical displacement from the Galactic plane toward the North Galactic Pole. We determined the Galactocentric distances ($R_{\text {GC}}$) by adopting a solar distance of 8.3 kpc to the Galactic center, consistent with the parameters established by^37^ . These distances play a crucial role in studying the structure of the Galaxy and identifying the signatures of the Milky Way’s spiral arms. The complete set of parameters derived for the clusters in this study is summarized in Table 2.

**Table 1:** Gaia astrometric solution and photometric properties for the identified massive members. Columns include position (RA, Dec), parallax ($\bar{\omega }$), proper motion components ($\mu ^{*}_{\alpha }, \mu _{\delta }$), the *Gaia* source identifier, estimated effective temperature ($T_{\textrm{eff}}$), derived mass, and the nominal spectral classification estimated from photometric temperatures (SpT).

RA (deg)	Dec (deg)	$\boldsymbol{\bar{\omega }}$ (mas)	$\boldsymbol{\mu _{\alpha }^{*}}$ (mas/yr)	$\boldsymbol{\mu _{\delta }}$ (mas/yr)	Source	$T_{\textrm{eff}}$ (k)	Mass $(M_{\odot })$	SpT
117.149	−26.981	0.220	−2.467	3.146	5601620626622013312	16036	2.931	B5
117.153	−26.988	0.200	−2.481	3.216	5601620630928384000	14680	2.601	B6
117.151	−26.986	0.202	−2.467	3.164	5601620630928384128	12228	2.282	B9
117.172	−26.964	0.247	−2.514	3.158	5601620768367314688	13183	5.088	B8
117.166	−26.960	0.202	−2.469	3.148	5601620768367317888	12992	2.517	B8
117.161	−26.952	0.214	−2.479	3.192	5601620832775307648	16854	3.287	B4
117.128	−26.949	0.190	−2.420	3.245	5601621627360796544	11617	2.683	B9
117.084	−26.945	0.225	−2.434	3.187	5601621764799762304	11916	2.112	B9
117.192	−26.866	0.214	−2.464	3.149	5601623929463163776	16236	3.081	B5

**Table 2:** Fundamental parameters of AH03_J0748-26.9 obtained from the present analysis compared with previous studies. The symbol “....” denotes parameters that were not calculated or reported by the respective references.

Parameter	This study	^12^	^38^
Cluster name	AH03_J0748-26.9	AH03_J0748-26.9	FSR_1315
*RA*(*deg*)	117.215 ± 0.01	117.155	117.159
*Dec*(*deg*)	−26.972 ± 0.005	−26.972	−26.974
*l* (*deg*)	243.226 ± 0.007	243.200	–
*b* (*deg*)	−0.582 ± 0.007	–	–
$\bar{\omega }$ (*mas*)	0.207 ± 0.005	*0.209± 0.033*	0.208±0.048
$\bar{\omega }_{corrected}$ (*mas*)	0.236 ± 0.005	–	–
$d_{\bar{\omega }}(kpc)$	4.834 ± 0.127	3.979	–
$d_{iso}(kpc)$	4.845 ± 0.056	–	–
$X_{\odot }(kpc)$	−10.226 ± 0.006	−9.916	–
$Y_{\odot }(kpc)$	−4.170 ± 0.003	−3.551	–
$Z_{\odot }(kpc)$	−0.021 ± 0.007	−0.0181	–
$R_{GC}(kpc)$	11.043 ± 0.004	–	–
*τ *	3.574	–	–
$M_{total}$ $(M_{\odot })$	290.603 ± 3.605	*411.325± 86.730*	–
$\mu _{\alpha }^{*}$ (*mas*/*yr*)	−2.176 ± 0.005	* - 2.465 ± 0.042 *	−2.435± 0.079
$\mu _{\delta }$ (*mas*/*yr*)	3.185 ± 0.006	*3.185± 0.051*	3.123±0.106
$m_{clSize}$	80	10	–
$m_{pts}$	10	10	–
*N* (*Stars*)	128	68	–
*logage* (*yr*)	8.06 ± 0.020	8.061	–
$T_{R}$ (*Myr*)	32.125	–	–
$\alpha _{\textrm{A}}$	* - 3.000 ± 0.605 *	–	–
$\alpha _{\textrm{B}}$	*1.596 ± 0.183*	–	–
$f_{b}$ $(Stars/degree^{2})$	26.561 ± 4.948	–	–
$f_{0}$ $(Stars/degree^{2})$	10812.064 ± 1552.062	–	–
$R_{50}(degree)$	0.084	0.034	0.08
${r_{c}}$ (*degree*)	0.098 ± 0.013	0.027	–
${r_{t}}$ (*degree*)	0.244 ± 0.015	0.146	–
*E(BP-RP)* (*mag*)	0.410 ± 0.050	–	–
*E(B-V)* (*mag*)	0.306 ± 0.037	–	–
$A_{V}$ (*mag*)	0.913 ± 0.111	1.036	–
$A_{G}$ (*mag*)	0.763 ± 0.093	–	–
*(m-M)* (*mag*)	13.427 ± 0.093	13.521	–
RV	69.745 ± 9.615	*79.360± 16.098*	–

## Estimation of mass function of AH03_J0748-26.9

The Initial Mass Function (IMF) describes the frequency distribution of stellar masses at birth. Given that a star’s mass is the primary driver of its physical characteristics and life cycle, the IMF serves as a fundamental framework for analyzing stellar systems. Because young clusters have undergone minimal member loss via dynamical interactions or stellar evolution, they serve as ideal laboratories for probing the IMF^26^. The IMF is conventionally formulated as a probability density function (PDF), representing the statistical likelihood of a star possessing a given mass at its inception^39^. In order to derive the masses of individual cluster members, synthetic stellar populations are modeled based on precisely defined fundamental parameters. A Monte Carlo simulation is utilized to derive final stellar masses and quantify their associated uncertainties by statistically comparing observed data with synthetic cluster models. The construction of synthetic clusters is predicated upon a comprehensive suite of theoretical isochrones. To facilitate the derivation of individual stellar masses, we generated a synthetic reference grid consisting of 10, 000 stars. It is important to clarify that this synthetic population does not represent the actual total population of AH03_J0748–26.9; rather, it serves as a dense, continuous lookup table in multi-dimensional photometric space. The large sample size of 10, 000 stars ensures high spatial resolution in the CMD, minimizing discretization errors and Monte Carlo noise when identifying the nearest synthetic neighbor for each of the 128 observed cluster members. A binary fraction of 50% is adopted, with companion masses derived from a uniform distribution ranging from zero to the mass of the primary component^40^. To ensure empirical realism, the synthetic clusters incorporate photometric uncertainties that align with the standardized Gaia definitions^41^. To establish a correspondence between empirical data and simulations, the magnitudes of each observed star are cross-referenced against the 10,000 synthetic counterparts within the model population. The synthetic star exhibiting the smallest magnitude deviation is identified by minimizing the Euclidean distance within the photometric space. This distance is calculated as the square root of the summed squared differences between the observed and synthetic magnitudes across all relevant bands.

$$ d_i = \min _{s \in S} \sqrt{ \sum _{j=1}^{m} \left( O_{ij} - S_{sj} \right) ^2 } $$

In this expression, $d_{i}$ denotes the minimum Euclidean distance between an observed star *O* and the set of *s* synthetic stars *S*, computed by summing the differences across *m* available magnitude bands. Following the identification of the nearest synthetic neighbor, the star is evaluated to ascertain its status as either an isolated object or a component of a binary system. Precise determination of the binary fraction is essential, given that binary systems—whether primordial or dynamically captured—can substantially bias the resulting mass function^27^. Should the identified synthetic counterpart be a binary system, the observed star is categorized accordingly, with the respective primary and secondary masses from the synthetic model assigned to that observation. Conversely, if the synthetic counterpart is identified as a single-star system, the mass of that individual synthetic star is attributed directly to the observed source. This procedure is iterated across multiple cycles, with each iteration producing a unique synthetic cluster through the stochastic variation of individual stellar members^40^. The definitive masses for observed stars and their companions are derived from the median of the sampled distribution, while the associated uncertainties are quantified by the standard deviation of these estimates. By minimizing Euclidean distances within the magnitude space, this methodology leverages synthetic companion masses to effectively populate the regions of the CMD where binary systems are expected to reside^40^.

The aggregate mass of the cluster is determined by integrating both the empirically observed stellar masses and the estimated masses of unobserved components, such as low-mass companions. To account for the unobserved mass fraction, specifically within the low-mass regime, the present-day mass function (MF) is first extrapolated from the distribution of empirically detected stellar masses. By integrating the mass function from the observational detection threshold down to a lower limit of $0.10\,M_{\odot }$, we account for the missing mass fraction. This extrapolation is based on the theoretical limits of the isochrones utilized in our analysis^42^. The mass function is represented by a two-part segmented linear model, which is optimized against the observed stellar mass distribution and subsequently extrapolated to the lower mass threshold.

The function is defined as

5 $$\begin{aligned} f(x) = {\left\{ \begin{array}{ll} \alpha _B\, x + b_1, & \text {for } M \le M_c, \\ \alpha _A\, x + b_2, & \text {for } M > M_c, \end{array}\right. } \end{aligned}$$

where $\alpha _{A}$ and $\alpha _{B}$ represent the slopes of the mass function (MF) for the low-mass and high-mass segments, respectively. The parameters $M_{C}$ denotes the transition mass—or ’break point’—separating the two segments, while $b_{1}$ and $b_{2}$ represent the respective intercepts for each linear regime. Within the fitting procedure, the slopes $\alpha _{A}$ and $\alpha _{B}$ are treated as free parameters, while the intercepts $b_{1}$ and $b_{2}$ are constrained to maintain functional continuity at the transition mass, satisfying the condition $f(M_{C}^{-})=f(M_{C}^{+})$. For the generation of this reference grid, individual masses were sampled according to the^43^ initial mass function. A nominal binary fraction of *50%* was adopted to populate the grid—a standard intermediate value used in synthetic CMD constructions to ensure both the single-star main sequence and the binary sequence are adequately represented (e.g.^44^). Companion masses were drawn from a uniform mass ratio distribution, ranging from *q = 0* to *q = 1* (where $q = M_{\textrm{secondary}} / M_{\textrm{primary}}$). Although observational studies frequently indicate non-uniform mass-ratio distributions (often peaking at low *q* or favoring equal-mass systems), the uniform distribution is employed here as a conservative prior. By maximizing the spread of the synthetic binary sequence across the CMD, this approach ensures that the reference grid fully encompasses the photometric scatter induced by unresolved binaries. This prevents systematic biases when assigning primary masses to observed stars whose photometry is altered by an unseen companion. This mean mass ratio is subsequently applied to the primary stellar masses within the unobserved regime to estimate the aggregate contribution of hidden companions. We assume that primary stars follow the mass function derived from the empirical data, utilizing the observed binary fraction ($F_{bin}$) to quantify the total mass contributed by binary systems. The total cluster mass is ultimately quantified using the expression:

$$ M_{T}=M_{O}+M_{un}(1+F_{bin}\times q) $$

where $M_{T}$ represents the aggregate mass, $M_{O}$ is the mass derived from observed stellar members, and $M_{un}$ denotes the mass obtained by integrating the MF for the unobserved population. Utilizing the cluster parameters derived from ASTECA, we generate a synthetic cluster population and employ a Monte Carlo method https://ocmass.streamlit.app/Monte Carlo Method to estimate the individual mass of each stellar member.

**Fig. 9:**
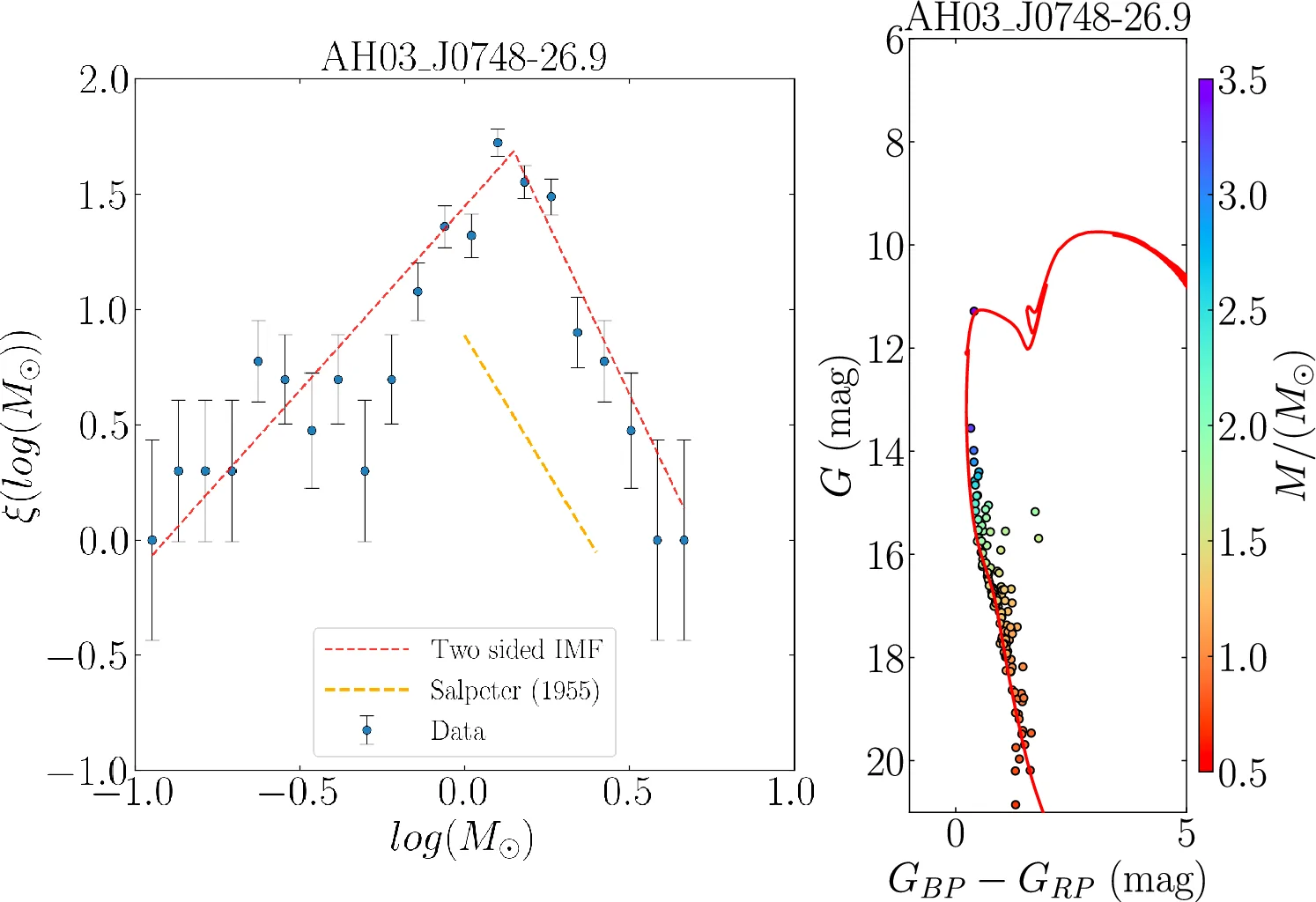
Left panel: The integrated mass function (MF) for AH03_J0748-26.9 is derived from a sample encompassing all stellar populations. For comparative purposes, the orange-dashed line represents the^52^ Initial Mass Function (IMF) slope. Right panel: The color-magnitude diagram (CMD) for the cluster under study, with the color bar indicating the stellar mass distribution.

The segmented linear function is subsequently fitted to the stellar mass histogram, with the bin width optimized according to Sturges’ rule^45^ to ensure an accurate representation of the distribution. Throughout this analysis, we define the mass function (MF) using the linear formalism

$$ \frac{dN}{dM} \propto M^{-\alpha }, $$

where *α * is the reported slope. For reference, the canonical Salpeter (1955) IMF in this convention corresponds to *α = 2.35*. Figure 9 illustrates the segmented linear fit (orange-dashed line) applied to the empirical mass distribution (green dots), highlighting the transition between different mass regimes and isochrone-fitted color-magnitude diagram for the cluster AH03_J0748-26.9.

Our analysis yields a segmented mass function with slopes of $\alpha _{\textrm{A}} = -3.000 \pm 0.605$ and $\alpha _{\textrm{B}} = 1.596 \pm 0.183$, with a transition mass $M_{\textrm{C}} = 0.150 \pm 0.025\,M_{\odot }$. According to the definition in Equation 5, the slope $\alpha _{\textrm{A}}$ describes the distribution of stars with masses $M > M_{\textrm{C}}$. This slope is significantly steeper than the canonical Salpeter (1955) value (*Γ = 1.35*, or $\alpha \approx 2.35$ in linear mass space, often represented as *-1.35* in logarithmic number-space). The observed steep slope ($\alpha _{\textrm{A}} \approx -3.0$) indicates a relative deficit of higher-mass stars compared to a standard IMF. Given the cluster’s age (*∼ 114.8* Myr) and derived relaxation time (*∼ 32* Myr), this steep slope is consistent with mass segregation and dynamical evolution, where higher-mass stars have settled into the core or been ejected, while the lower-mass population remains more extended or depleted in the outer regions surveyed.

Our derived steep slope ($\alpha _A \approx -3.0$) is significantly steeper than the Salpeter value (*α = 2.35*). This deviation points to a relative deficit of higher-mass stars compared to the canonical IMF, consistent with significant dynamical evolution. A comparison with recent *Gaia*-based studies reinforces this interpretation. For example, Bisht et al. (2020) analyzed four open clusters (Czernik 14, Haffner 14, Haffner 17, and King 10) and found mass function slopes ranging from *x = 1.27* to 1.38 in the $\frac{dN}{dM} \propto M^{-(1+x)}$ formalism. Converting to our convention (*α = 1+x*), their slopes correspond to $\alpha \approx 2.27-2.38$, which are in excellent agreement with the Salpeter value. Those clusters, with ages of *45-570* Myr and relaxation times generally exceeding their ages (Bisht et al., 2020^46^), are not yet dynamically relaxed, thus retaining a MF slope close to the initial Salpeter law. In contrast, Belwal et al. (2025) studied six intermediate-age clusters (ages *0.2-2* Gyr) and reported MF slopes (*α *) in the range *0.96-1.19* (converted to the $\frac{dN}{dM}$ formalism), which are significantly flatter than Salpeter. They attributed this flattening to the preferential loss of low-mass stars due to dynamical evolution and tidal interactions with the Galactic disk. Our derived slope for the high-mass regime ($\alpha _A \approx -3.0$) is steeper than Salpeter, which is complementary to the flattening observed by Belwal et al. (2025)^47^. The difference arises because our analysis focuses on the high-mass end ($M > 0.15\,M_{\odot }$) of the present-day MF in a dynamically relaxed cluster ($\tau = \textrm{age}/T_R \approx 3.6 > 1$), where mass segregation has concentrated massive stars in the core (which we sample) and dynamical evolution has depleted the intermediate-mass population relative to a Salpeter law. Taken together, these comparisons demonstrate that the shape of the MF in open clusters is highly sensitive to the interplay between age, relaxation time, and dynamical state, with AH03_J0748-26.9 representing an advanced stage of dynamical processing. The total cluster mass was estimated by integrating the observed mass function and extrapolating the contribution from unobserved low-mass stars and unresolved binary systems. We adopted a binary fraction of $f_{\textrm{b}} \approx 0.70$ (see^48,49^). This high binary fraction is supported by the cluster’s high concentration parameter (*c = 0.396*) and its relatively young age ($\log t=8.06 \pm 0.020$). At this evolutionary stage, dynamical disruption has not yet significantly depleted the primordial binary population, a finding consistent with observations in similar intermediate-age open clusters (e.g.^50^). The cluster hosts a significant population of photometrically identified B-type candidates (Table 1); extensive spectroscopic surveys have demonstrated that massive stars exhibit a significantly higher multiplicity fraction, often exceeding 60–*70%* (^44^). While the average binary fraction for open clusters typically ranges from 30–*50%* (^51^), the presence of massive stars and the cluster’s concentration supports the adoption of a higher value for this system. To assess the sensitivity of our mass estimate to this assumption, we performed the calculation using binary fractions of $f_{\textrm{b}} = 0.5$ and $f_{\textrm{b}} = 0.9$. The resulting total masses were $265\,M_{\odot }$ and $315\,M_{\odot }$, respectively. This indicates that the total mass estimate has an uncertainty of roughly $\pm 10\%$ due to the binary fraction assumption, which is smaller than the statistical uncertainties of the fit. Thus, the derived mass of $290.603 \pm 3.605\,M_{\odot }$ is robust.

For the high-mass regime ($1.107<M/M_{\odot }<5.088$), we find a Mass Function (MF) slope that is consistent, within 1*σ * uncertainties, with the canonical^52^ Initial Mass Function (IMF) value of *Γ =-1.35*. In contrast to the high-mass regime, the MF slope for stars in the range $0.102<M/M_{\odot }<1.407$ exhibits a significant departure from the^52^ value. The disparity between the high-mass and low-mass MF slopes is consistent with mass segregation within AH03_J0748-26.9, providing evidence for, rather than definitive proof of, significant dynamical evolution in this cluster; an equally steep slope could in principle also be shaped by stochastic sampling in a modestly populated cluster, so we regard this interpretation as the most plausible explanation supported by the available data rather than a firm conclusion.

We emphasize that the low-mass segment of the derived MF ($M<0.15\,M_{\odot }$, slope $\alpha _B$) is not directly resolved star-by-star from *Gaia* photometry alone. Near the faint end of the observed CMD, *Gaia* DR3 completeness declines and photometric uncertainties grow, so only a limited number of the lowest-mass candidate members satisfy our membership and photometric quality cuts. The mass assigned to each observed star is obtained by matching its position in the CMD against the synthetic reference grid, which was itself populated by sampling the^43^ IMF; consequently, the shape of the MF below $M_C$ is constrained jointly by the (comparatively sparse) observed low-mass members and by the mass–luminosity relation and priors built into the synthetic model and underlying isochrones, rather than by direct, densely sampled *Gaia* detections as is the case at higher masses. This means $\alpha _B$ should be regarded as a model-dependent extrapolation calibrated against the available faint members, and is correspondingly more uncertain than $\alpha _A$. Incompleteness could bias this slope in either direction: if faint, low-mass members are preferentially missed near the detection limit, the observed low-mass counts could be artificially suppressed, flattening or even inverting the apparent slope; conversely, if photometric scatter scatters some higher-mass stars into the low-mass bins, the slope could be artificially steepened.

We calculated the dynamical relaxation time ($T_{R}$), denoting the duration required for individual stars to exchange sufficient energy to attain a velocity distribution conforming to Maxwellian equilibrium. We employed the methodology outlined by^53^ for estimating this dynamical relaxation time ($T_{R}$) as

6 $$\begin{aligned} T_{R}=\frac{8.9\times 10^{5}\times \sqrt{N}\times R_{h}^{1.5}}{\sqrt{\overline{m}}\times \log \left( 0.4\times N\right) } \end{aligned}$$

Where *N* represents the total number of stars within the cluster, $R_{h}$ denotes the half-mass radius of the star cluster, and $\overline{m}$ signifies the average stellar mass present in the cluster. The mean mass $(\overline{m})$ has been determined as $2.27\pm 0.028\,M_{\odot }$ for AH03_J0748-26.9. In the computation of $R_{h}$, we initially aggregate the individual masses of cluster members and subsequently derive both the mean stellar mass and the total mass of the cluster. By identifying the radius encompassing *50%* of the total mass of the cluster, we determine the value of $R_{h}$, thereby facilitating the calculation of $T_{R}$. The dynamical state is quantified by the parameter $\tau = \text {age} / T_R$, where *τ > 1* indicates a dynamically relaxed system (Spitzer & Hart 1971)^53^. For AH03_J0748-26.9, $\tau = 114.815 / 32.125 \approx 3.57 > 1$, indicating that the cluster has reached dynamical relaxation. Table 2 summarizes all derived parameters.

### Investigating the orbital dynamics of AH03_J0748-26.9

**Fig. 10:**
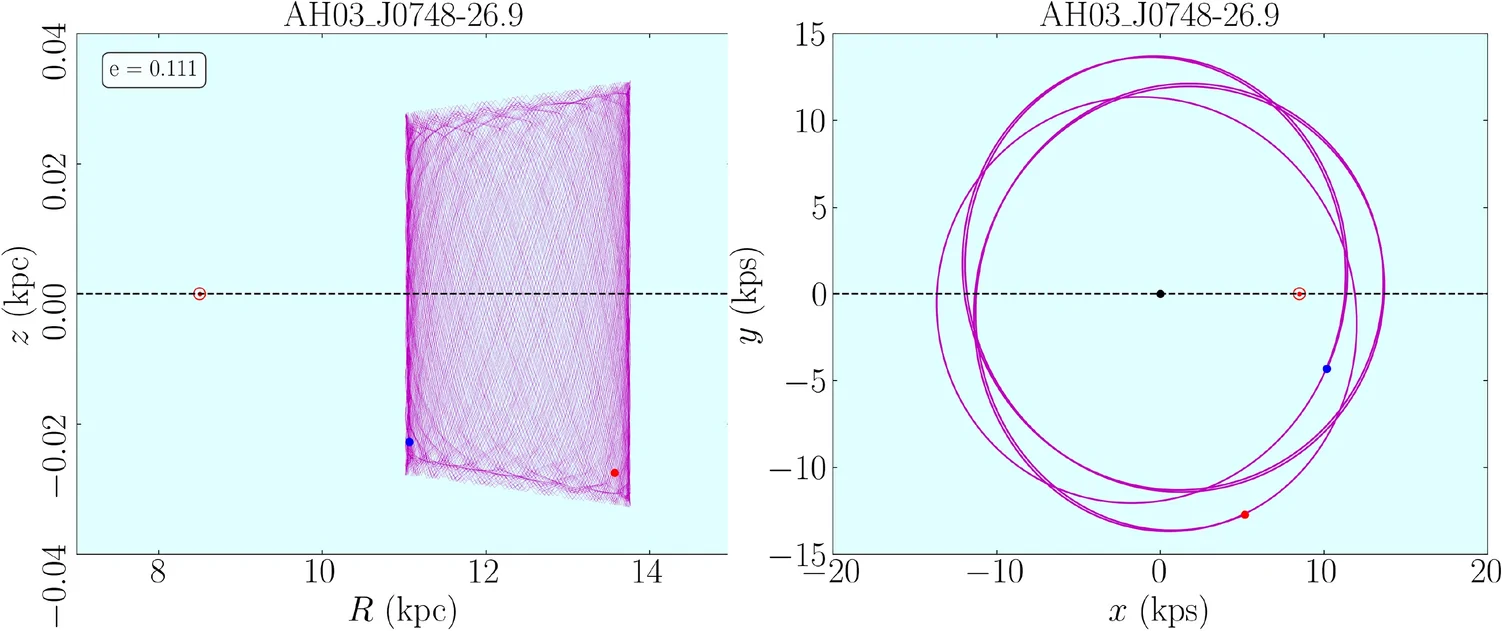
Galactic orbits of AH03_J0748-26.9 computed using the galpy library. In both panels, the red dot denotes the cluster’s current position, while the blue dot marks the starting point of the backward integration (the birth location). The Galactic center and the Sun are represented by a black dot and a standard solar symbol (*⊙ *), respectively.

Open clusters act as essential laboratories for investigating Galactic structure and stellar dynamics^54^. Determining birth radii and galactic distribution via kinematics and dynamics sheds light on cluster formation and evolution, ultimately enhancing our understanding of star formation processes in the Milky Way^27^. The distribution of open clusters in the solar neighborhood can be analyzed by determining fundamental parameters such as radial velocities, distances, and ages. This illuminates their origins and present locations within the Galactic plane^55^. We simulated the orbital integrals of clusters within the Galactic potential field using the {$\texttt {galpy}$} http://github.com/jobovy/galpy package^56^. The model utilizes an axisymmetric Milky Way potential consisting of three distinct components: a spherical bulge^56^, a Galactic disk^57^, and a spherical dark-matter halo^58^. For the orbit integration of AH03_J0748-26.9, we adopt the final distance $d_{\textrm{iso}} = 4.845 \pm 0.056$ kpc derived from the best-fit PARSEC isochrone (Sect. “Galactic distribution, evolutionary age, and distance estimates”). This value is also consistent with the Gaia parallax distance $d_{\varpi }=4.834\pm 0.127$ kpc and the Bayesian geometric distance (*4.839 ± 0.102* kpc). All subsequent orbital calculations (apocenter, pericenter, eccentricity, and space velocities) are therefore based on this single, consistently adopted distance. In addition to the astrometric data, we adopted a mean radial velocity of *RV=69.745± 9.615* km s$^{-1}$ for AH03_J0748-26.9. Our orbital calculations for AH03_J0748-26.9 were conducted under the assumption of a Galactocentric solar distance ${R_{gc}=8.3}$ kpc, an orbital rotation velocity $V_{rot}=220$ km s$^{-1}$^56,59^, and a solar offset above the Galactic midplane of $Z_{\odot }=25\pm 5$ pc^60^. The integrated orbit of the open cluster AH03_J0748-26.9 within the Galactic potential is shown in Fig. 10. The left panel of Fig. 10 illustrates the meridional projection of the orbit of AH03_J0748-26.9. The Galactocentric radius $R_{GC}$ is plotted on the abscissa against the vertical distance from the Galactic plane *Z* on the ordinate. To estimate the likely birth radius of AH03_J0748-26.9, we performed a backward orbit integration over a duration of 114.815 Myr, corresponding to the cluster’s estimated age, following the methodology of^61^. This model accounts for uncertainties in the Galactic potential as well as observational errors in distance, radial velocity, and proper-motion components.

The following key parameters were derived from the orbital integration of AH03_J0748-26.9: Based on the calculated apocenter ($R_{\textrm{apo}}=13.767$ kpc) and pericenter ($R_{\textrm{peri}}=11.011$ kpc) distances, we determined the orbital eccentricity to be *e=0.111*, using the relation:

$$ e=\frac{R_{\textrm{apocenter}}-R_{\textrm{pericenter}}}{R_{\textrm{apocenter}}+R_{\textrm{pericenter}}}. $$

Regarding its kinematics, AH03_J0748-26.9 exhibits Galactic space velocity components of $U=9.28\text { km s}^{-1}$, $V=231.94\text { km s}^{-1}$, and $W=0.79\text { km s}^{-1}$. For the transformation to the Local Standard of Rest (LSR), we adopted the solar motion components derived from high-probability thin-disk stars in^62^: $(U, V, W)_{\odot } = (8.50 \pm 0.21,\ 13.38 \pm 0.53,\ 6.49 \pm 0.20)\,\mathrm {km\,s^{-1}},$ where *U* is directed toward the Galactic Center, *V* in the direction of Galactic rotation, and *W* toward the North Galactic Pole. Applying these corrections yields the LSR-corrected space velocity components $(U,V,W)_{\textrm{LSR}}=(79.63 \pm 0.21,-35.77 \pm 0.53,-12.95 \pm 0.20)\text { km s}^{-1}$, corresponding to a total space velocity of $S_{\textrm{LSR}}=88.25 \pm 0.29\text { km s}^{-1}$ for AH03_J0748-26.9.

## Conclusion

This study provides a comprehensive analysis of the open cluster AH03_J0748-26.9, employing Gaia DR3 astrometric and photometric data to characterize its fundamental properties. By applying a stringent membership criterion (*P>50%*), we identified 128 probable members for AH03_J0748-26.9, ensuring robust cluster characterization. The primary findings of our analysis are summarized below:

1. The radial density profile, fitted with a King model, yields a tidal radius of $r_{t}=0.244 \pm 0.015$ deg and a core radius of ${r_{c}}=0.098 \pm 0.013$ deg for AH03_J0748-26.9. The cluster under investigation exhibited mean proper motions of $(-2.176\pm 0.005,3.185\pm 0.006)\text { mas yr}^{-1}$. A median parallax of *0.207 ± 0.005* mas translates to a heliocentric distance of $d_{\varpi }=4.834\pm 0.127$ kpc. The best-fit PARSEC isochrone yields $d_{\textrm{iso}} = 4.845 \pm 0.056$ kpc, and the Bayesian geometric distance (Bailer-Jones et al. 2021)^33^ gives *4.839 ± 0.102* kpc. We adopt $d_{\textrm{iso}} = 4.845 \pm 0.056$ kpc as the final distance for all analyses, given its higher precision and consistency with the CMD morphology.
2. By fitting PARSEC isochrones, we determined a distance modulus of *13.427 ± 0.093* mag and a logarithmic age of ($\log t=8.06 \pm 0.020$) (114.815 Myr). The calculated relaxation time of 32.125 Myr suggests that AH03_J0748-26.9 is a dynamically relaxed system.
3. The presence of massive B-type stars and a mean age of 114.815 Myr characterize AH03_J0748-26.9 as a young cluster. The alignment between Gaia-derived astrometry and the Color-Magnitude Diagram supports the view that this cluster serves as a primary empirical benchmark for studying the Initial Mass Function and early stellar evolution.
4. We estimated the mass function for AH03_J0748-26.9 across the range $0.102-5.088\,M_{\odot }$, obtaining MF slopes of $\alpha _A = -3.000 \pm 0.605$ and $\alpha _B = 1.596 \pm 0.183$ with a characteristic mass $M_C = 0.150 \pm 0.025\,M_{\odot }$, following the convention $\frac{dN}{dM} \propto M^{-\alpha }$. The steep high-mass slope ($\alpha _A = -3.000$) deviates significantly from the Salpeter (1955) value (*α = 2.35*) and from the near-Salpeter slopes found in less dynamically evolved clusters^46^, while being complementary to the flatter slopes reported for other dynamically processed systems^47^. This suggests that AH03_J0748-26.9 has likely undergone significant dynamical evolution and mass segregation, though the steep slope should be interpreted with caution given the modest member sample and the uncertainties in the mass function fitting at the high-mass end. We similarly note that the low-mass slope ($\alpha _B$) below $M_C$ is constrained jointly by a comparatively sparse set of observed faint members and by the synthetic population model used to assign masses, and is therefore sensitive to *Gaia* completeness near the detection limit; this slope should likewise be treated as indicative rather than definitive. Integration of the best-fit mass function yields a total cluster mass of $290.603 \pm 3.605\,M_{\odot }$, assuming a binary fraction of *∼ 0.70*.
5. Based on 128 identified member stars with radial velocity measurements, we obtained a mean value of $69.745\pm 9.615\ \text {km}\ \text {s}^{-1}$. By integrating these data with proper motion measurements, we utilized the galpy Python package to derive the orbital parameters for AH03_J0748-26.9.

## Data Availability

The astrometric and photometric data used in this study are publicly available from the Gaia Data Release 3 (DR3) archive at the European Space Agency (https://gea.esac.esa.int/archive/). The specific data queries for AH03_J0748-26.9 were performed using the astroquery.gaia Python module. The Digitized Sky Survey (DSS) finding chart is available from the Space Telescope Science Institute (https://archive.stsci.edu/cgi-bin/dss_form). Additional cluster parameters and comparison data from15 are available from their published catalog. The Python codes used for HDBSCAN clustering, isochrone fitting, and orbital analysis with galpy are available from the corresponding author upon reasonable request.
